# Supplementary material for: Biobank-scale genetic characterization of Alzheimer’s disease and related dementias across diverse ancestries
Source: Nat Commun. 2025 Aug 14;16:7554. doi: 10.1038/s41467-025-62108-y (PMC12354765; doi:10.1038/s41467-025-62108-y)
Supplement: Supplementary file 1 — Supplementary Information [file 41467_2025_62108_MOESM1_ESM.pdf]

Supplementary Information

Biobank-scale genetic characterization of Alzheimer's disease and related dementias across diverse ancestries

Marzieh Khani[1], Fulya Akçimen[2#], Spencer M. Grant[1#], Suleyman Can Akerman[3, 4], Paul Suhwan Lee[1], Faraz Faghri[1,5], Hampton Leonard[1,5] , Jonggeol Jeffrey Kim[1], Mary B. Makarious[1,5], Mathew J. Koretsky[1,5], Jeffrey D Rothstein[3,4], Cornelis Blauwendraat[1,2], Mike A. Nalls[1,5], Andrew Singleton[1], Sara Bandres-Ciga[1,*]

Affiliations:

[1] Center for Alzheimer's and Related Dementias (CARD), National Institute on Aging and National Institute of Neurological Disorders and Stroke, National Institutes of Health, Bethesda, MD 20892, USA

[2] Molecular Genetics Section, Laboratory of Neurogenetics, National Institute on Aging, National Institutes of Health, Bethesda, MD 20892, USA

[3] Brain Science Institute, Johns Hopkins University School of Medicine, Baltimore, MD, 21205, USA

[4] Department of Neurology, Johns Hopkins University School of Medicine, Baltimore, MD 21205, USA

[5] Data Tecnica LLC, Washington, DC 20037, USA

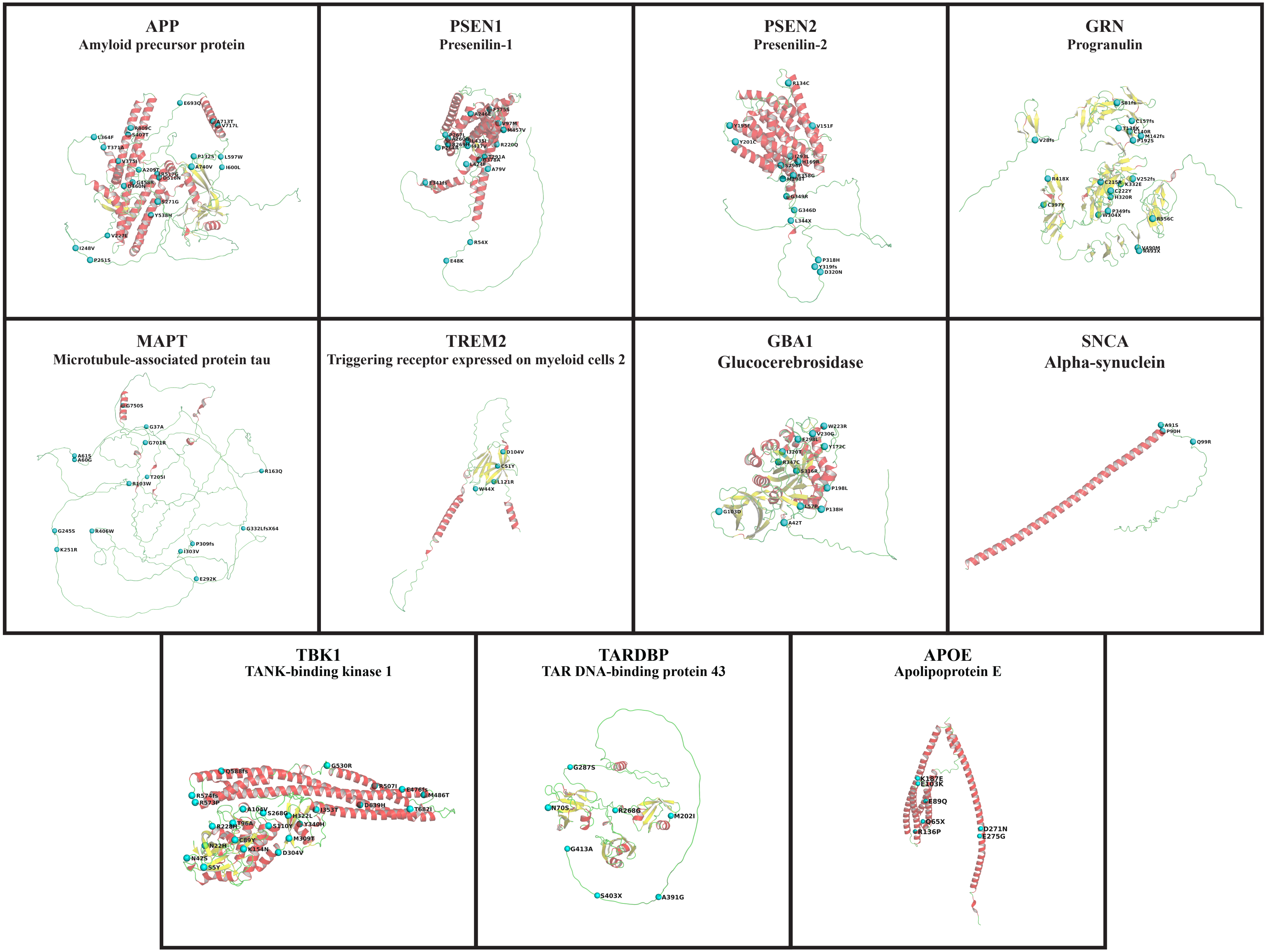

Supplementary Figure 1 - Mutation sites from identified genetic variants mapped on the predicted protein structures encoded by genes associated with AD/ADRDs.

The predicted protein structures encoded by 11 genes associated with AD/ADRDs (APP, PSEN1, PSEN2, TREM2, GBA1, GRN, MAPT, SNCA, TBK1, TARDBP, and APOE) were obtained from the EMBL AlphaFold Protein Structure Database to ensure that all of the residues were present in each protein structure. PyMOL v. 2.6.0 was used to represent the protein structures and their associated mutation sites from identified genetic variants. The yellow color shows beta sheets, the red color shows alpha helices, and the green color shows connecting loops and turns.

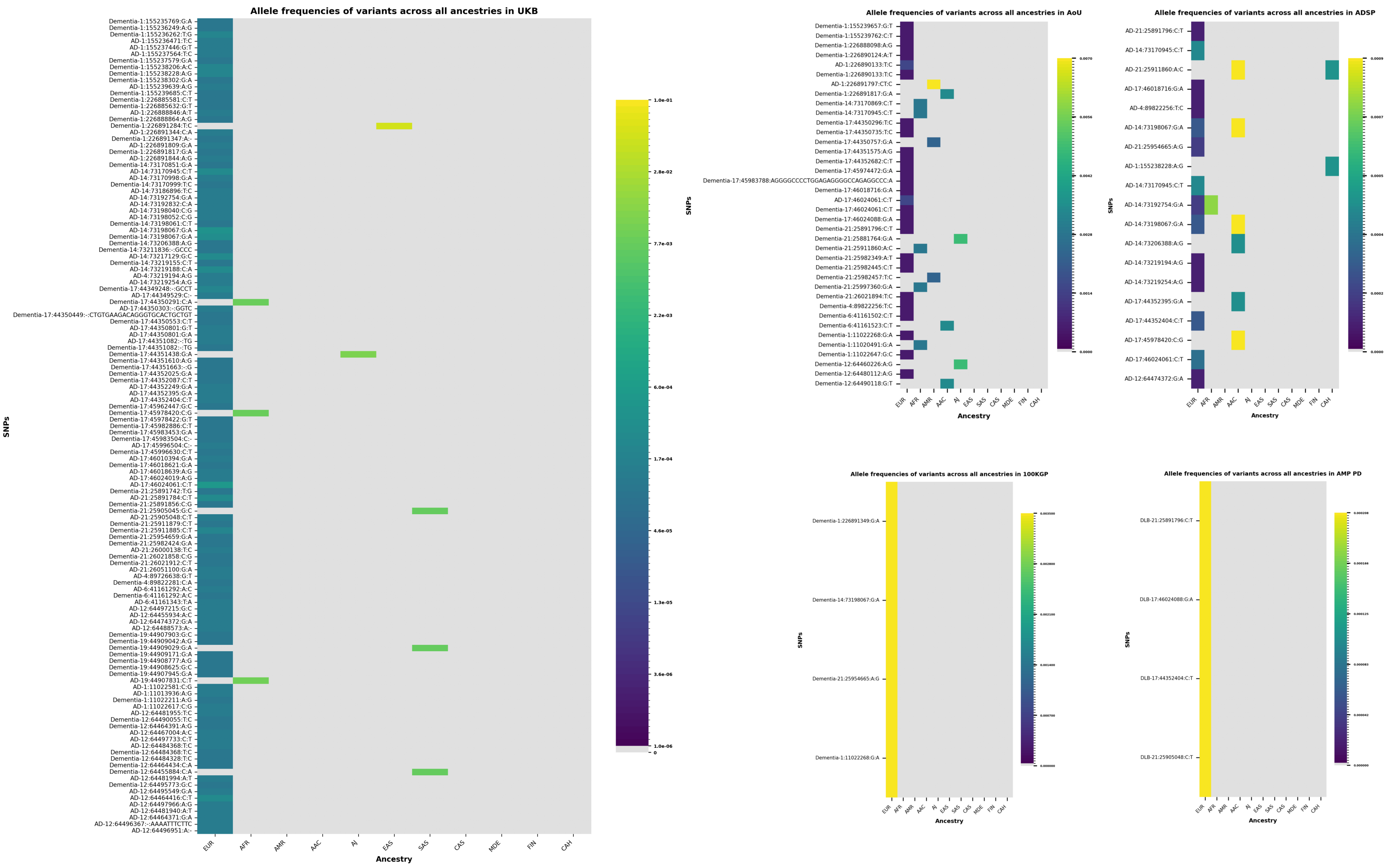

Supplementary Figure 2 - Heatmaps showing the frequencies of all identified variants in the discovery and replication phases across all ancestries in each biobank.

A

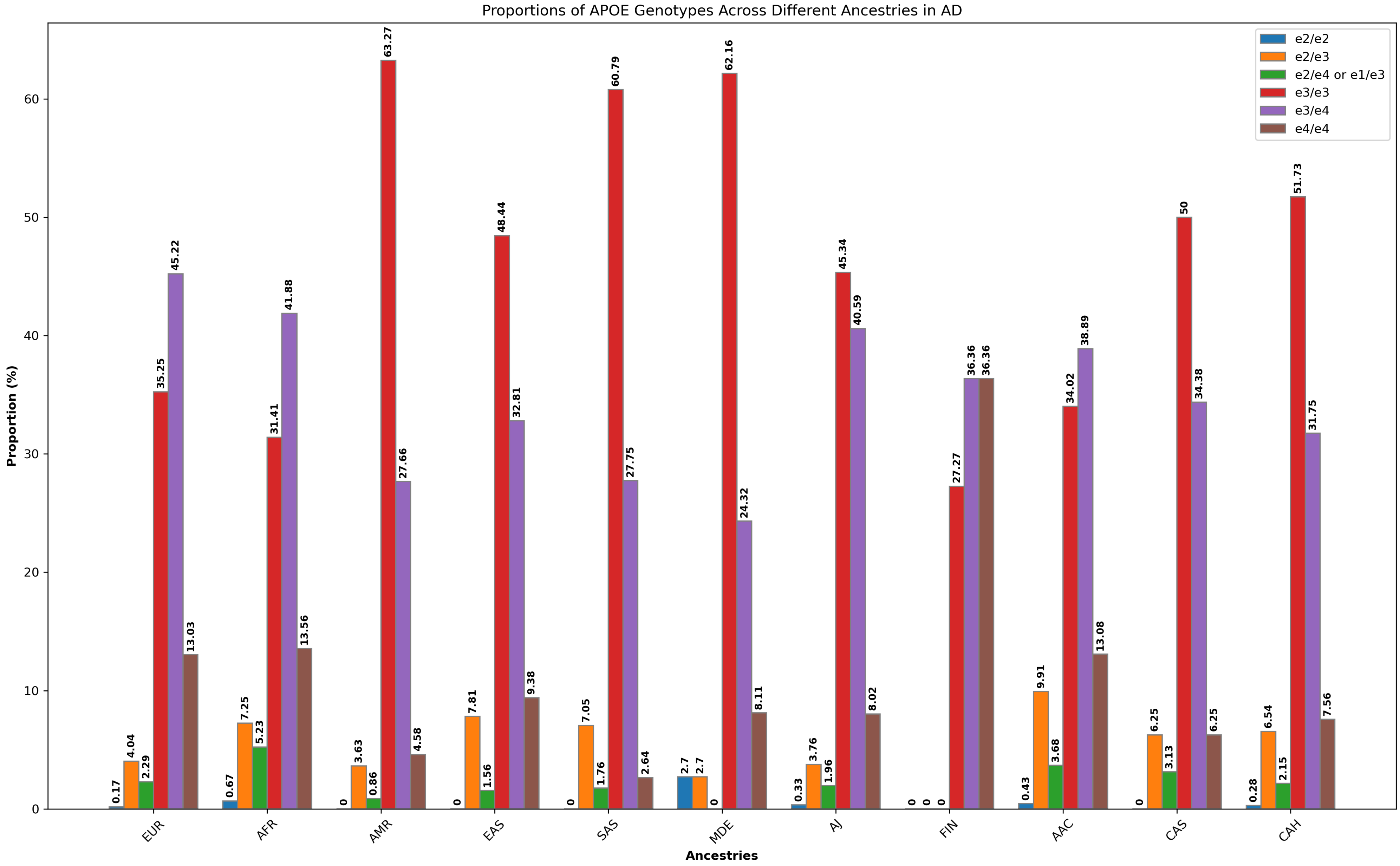

Supplementary Figure 3 - Proportion of APOE genotypes in (A) Alzheimer's disease, (B) related dementias, and (C) controls across 11 genetic ancestries.

Unknown genotypes and those absent across all ancestries were excluded from the analysis. Genotypes and ancestries not available in the 100KGP were also excluded.

B

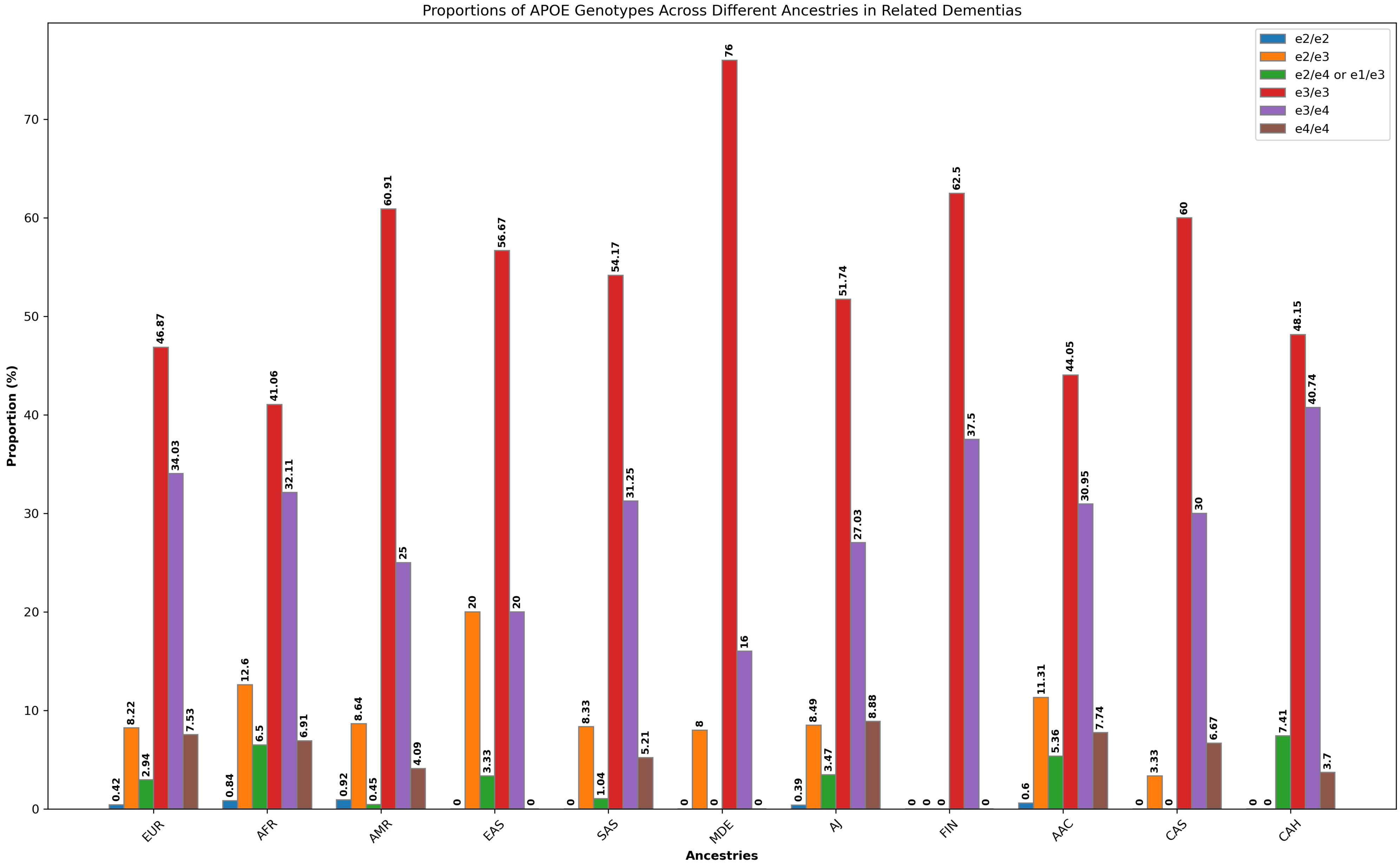

Supplementary Figure 3 - Proportion of APOE genotypes in (A) Alzheimer's disease, (B) related dementias, and (C) controls across 11 genetic ancestries.

Unknown genotypes and those absent across all ancestries were excluded from the analysis. Genotypes and ancestries not available in the 100KGP were also excluded.

C

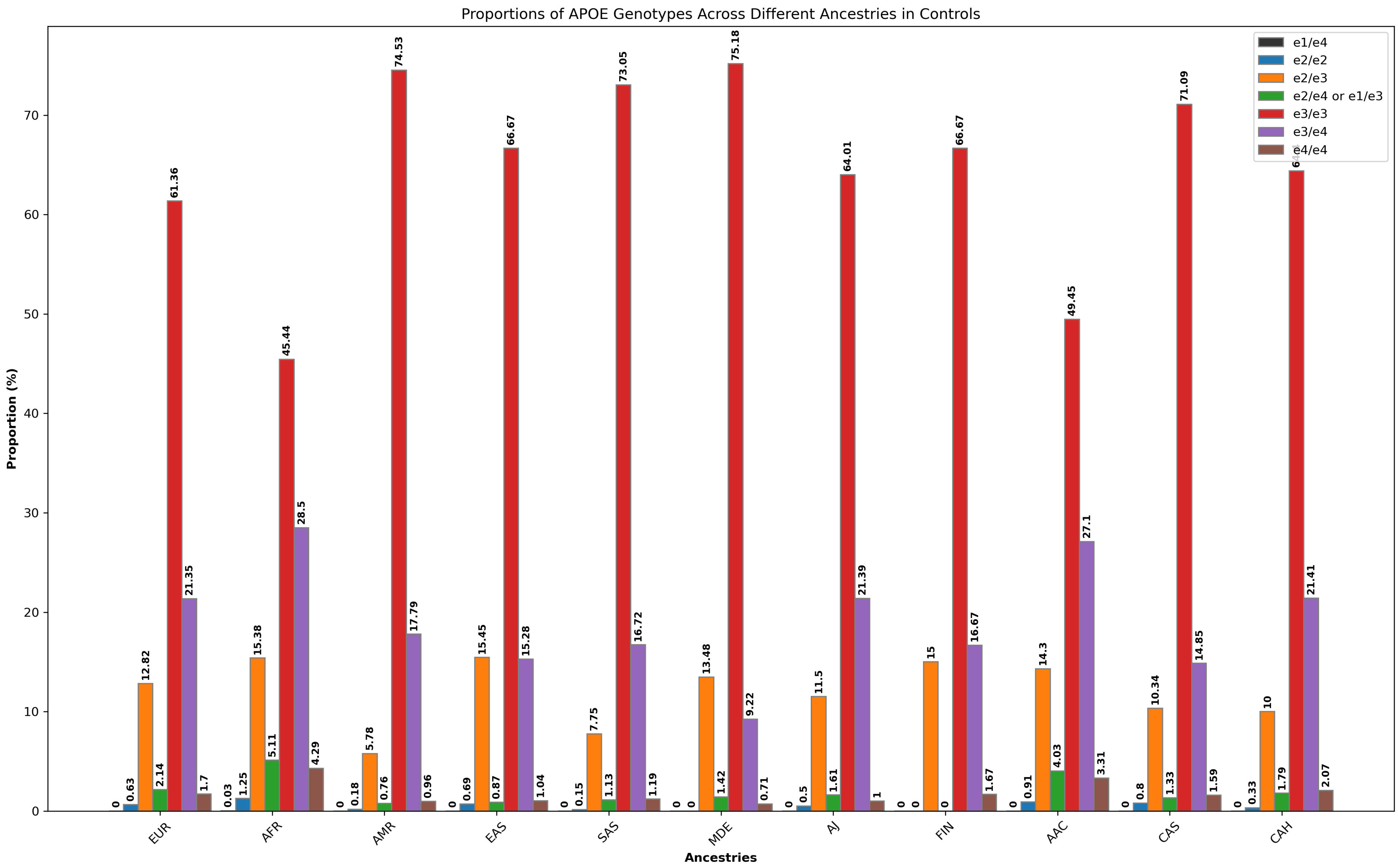

Supplementary Figure 3 - Proportion of APOE genotypes in (A) Alzheimer's disease, (B) related dementias, and (C) controls across 11 genetic ancestries.

Unknown genotypes and those absent across all ancestries were excluded from the analysis. Genotypes and ancestries not available in the 100KGP were also excluded.

A

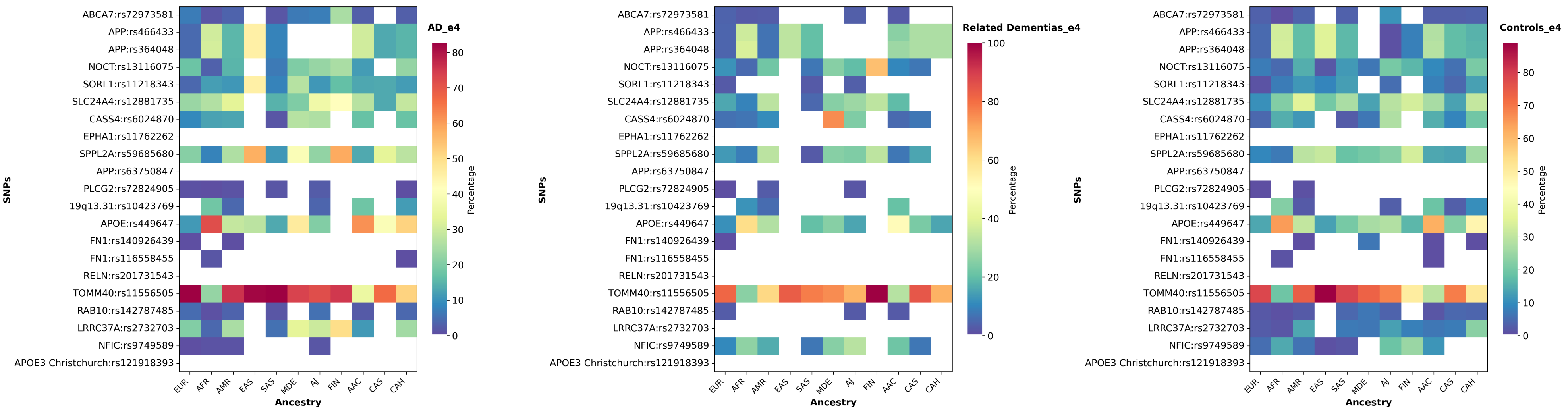

Supplementary Figure 4 - Proportions of individuals carrying both APOE ε4 or APOE ε4/ε4 genotypes and protective or disease-modifying variants across 11 genetic ancestries in Alzheimer's disease, related dementias, and controls in all datasets.

Supplementary Figures 4A, 4C and 4E represent SNP distribution within each cohort, and Supplementary Figures 4B, 4D, and 4F represent SNP distribution between cohorts. The total number of ε4 carriers per ancestry were used to generate 4A and 4B. The total populations of each ancestry were used to generate 4C and 4D. The total numbers of ε4/ε4 carriers for each ancestry were used to generate 4E and 4F. Supplementary Figures 4B, 4D, and 4F show allele frequency ratios (AD-to-Control, left; Related dementias-to-Control, right) among APOE ε4 and APOE ε4/ε4 carriers for each of the candidate protective or disease-modifying variant, per ancestry. Warmer colors represent higher frequencies in cases versus controls, while cooler colors represent higher frequencies in controls versus cases, with dark blue (N/A) representing variants not present in either cases or controls. 0 values in cases and controls are shown in white.

B

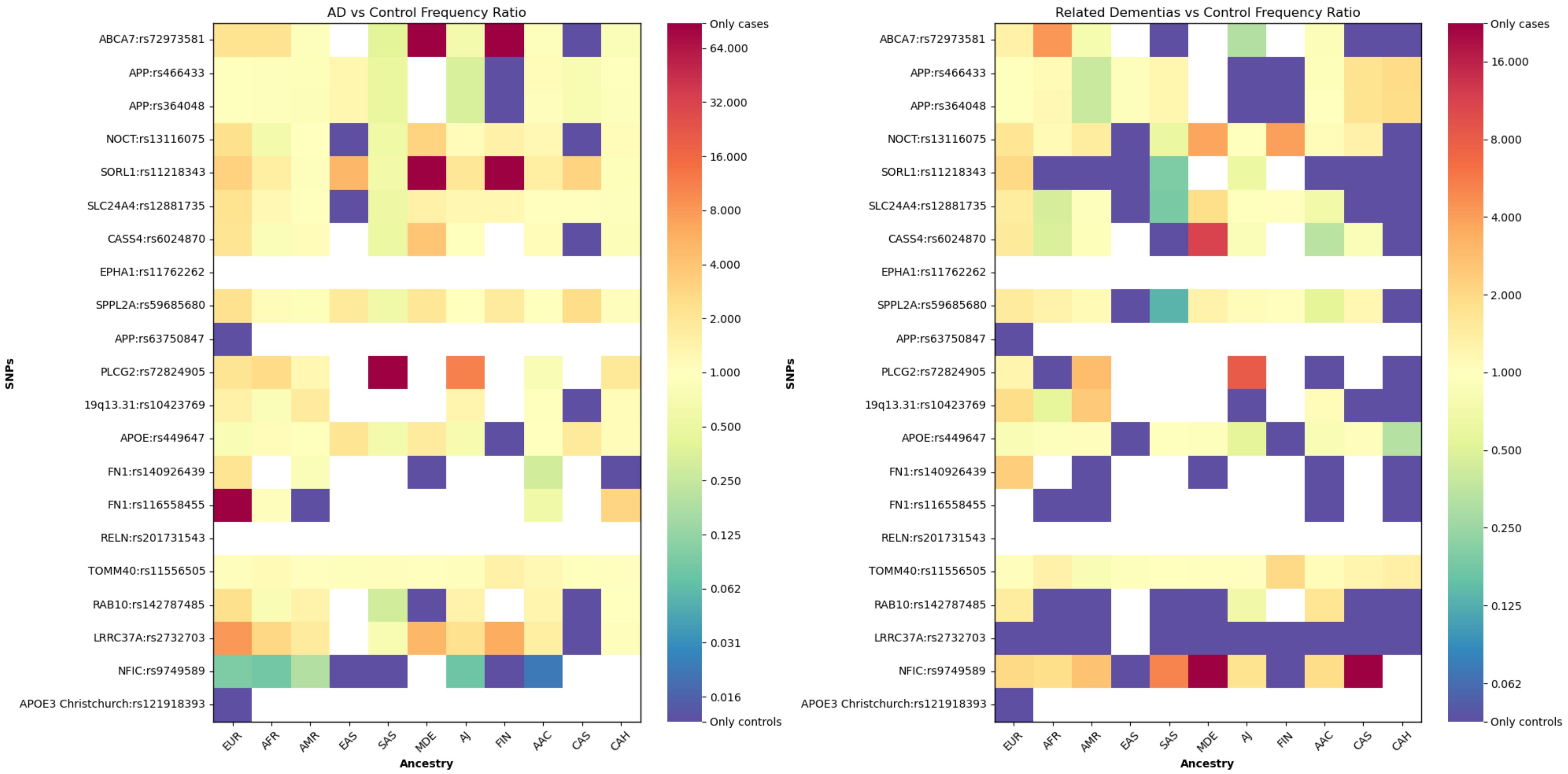

Supplementary Figure 4 - Proportions of individuals carrying both APOE ε4 or APOE ε4/ε4 genotypes and protective or disease-modifying variants across 11 genetic ancestries in Alzheimer's disease, related dementias, and controls in all datasets.

Supplementary Figures 4A, 4C and 4E represent SNP distribution within each cohort, and Supplementary Figures 4B, 4D, and 4F represent SNP distribution between cohorts. The total number of ε4 carriers per ancestry were used to generate 4A and 4B. The total populations of each ancestry were used to generate 4C and 4D. The total numbers of ε4/ε4 carriers for each ancestry were used to generate 4E and 4F. Supplementary Figures 4B, 4D, and 4F show allele frequency ratios (AD-to-Control, left; Related dementias-to-Control, right) among APOE ε4 and APOE ε4/ε4 carriers for each of the candidate protective or disease-modifying variant, per ancestry. Warmer colors represent higher frequencies in cases versus controls, while cooler colors represent higher frequencies in controls versus cases, with dark blue (N/A) representing variants not present in either cases or controls. 0 values in cases and controls are shown in white.

C

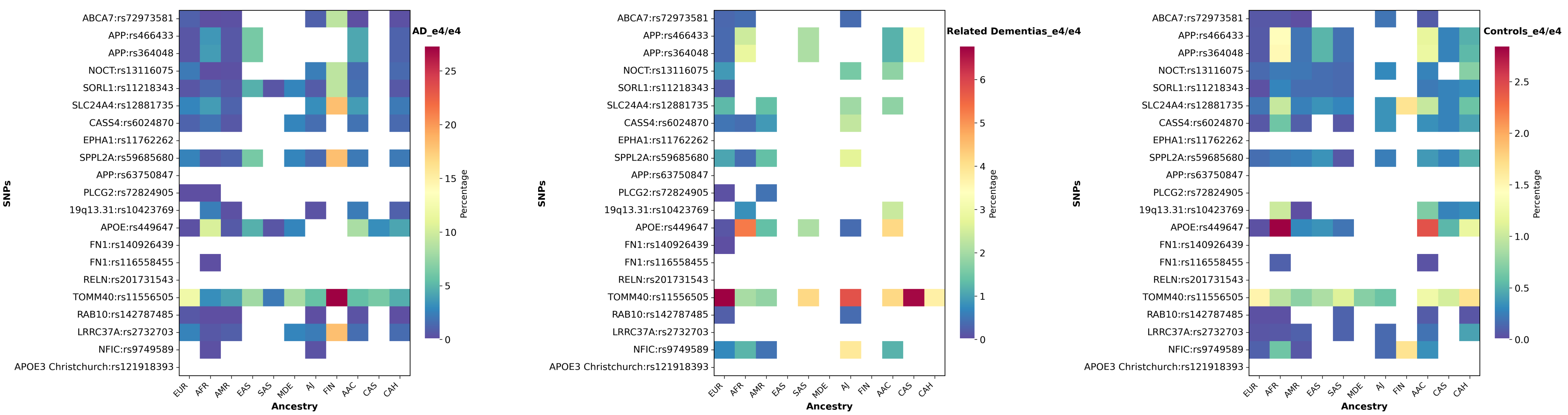

Supplementary Figure 4 - Proportions of individuals carrying both APOE ε4 or APOE ε4/ε4 genotypes and protective or disease-modifying variants across 11 genetic ancestries in Alzheimer's disease, related dementias, and controls in all datasets.

Supplementary Figures 4A, 4C and 4E represent SNP distribution within each cohort, and Supplementary Figures 4B, 4D, and 4F represent SNP distribution between cohorts. The total number of ε4 carriers per ancestry were used to generate 4A and 4B. The total populations of each ancestry were used to generate 4C and 4D. The total numbers of ε4/ε4 carriers for each ancestry were used to generate 4E and 4F. Supplementary Figures 4B, 4D, and 4F show allele frequency ratios (AD-to-Control, left; Related dementias-to-Control, right) among APOE ε4 and APOE ε4/ε4 carriers for each of the candidate protective or disease-modifying variant, per ancestry. Warmer colors represent higher frequencies in cases versus controls, while cooler colors represent higher frequencies in controls versus cases, with dark blue (N/A) representing variants not present in either cases or controls. 0 values in cases and controls are shown in white.

D

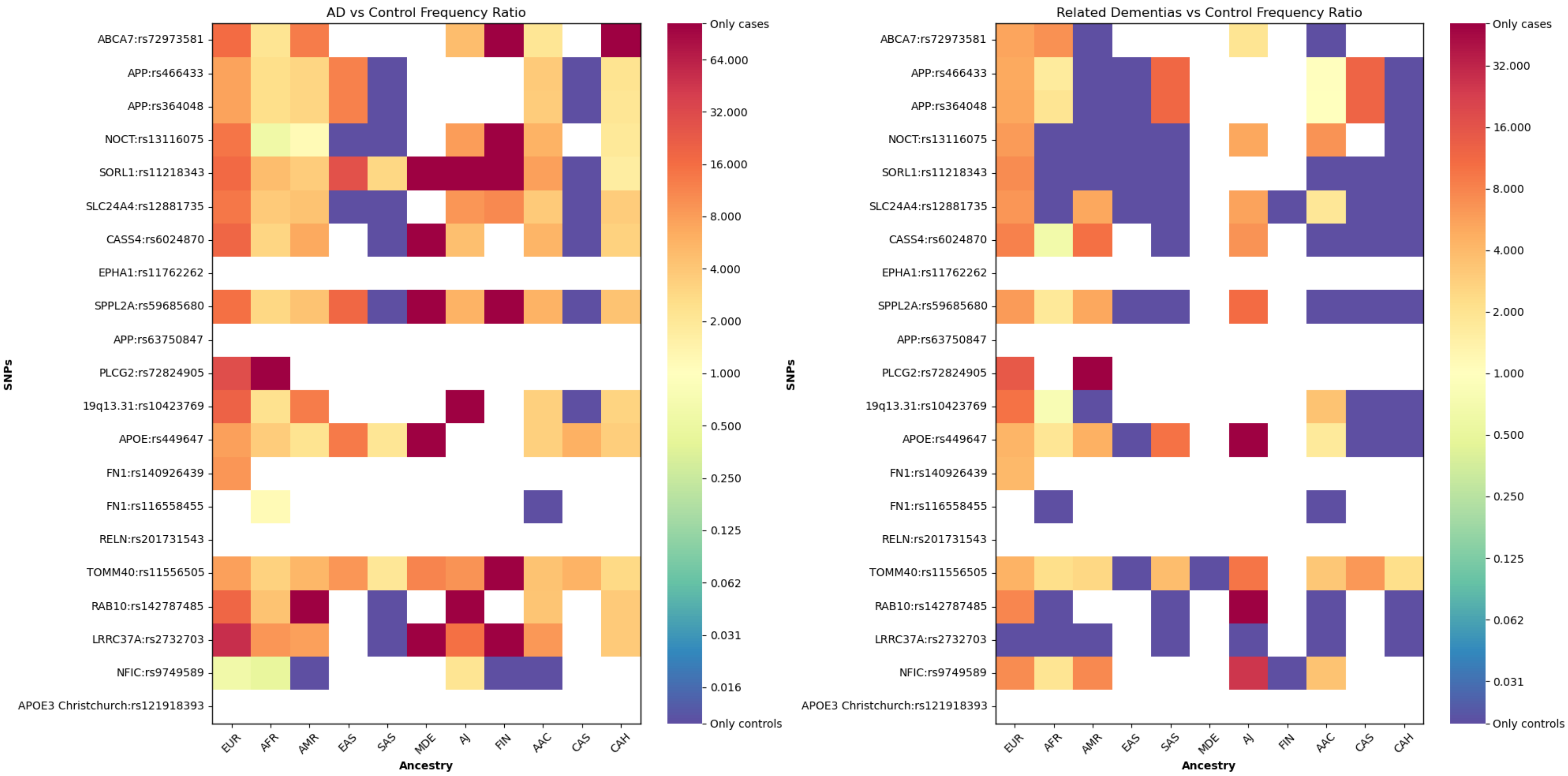

Supplementary Figure 4 - Proportions of individuals carrying both APOE ε4 or APOE ε4/ε4 genotypes and protective or disease-modifying variants across 11 genetic ancestries in Alzheimer's disease, related dementias, and controls in all datasets.

Supplementary Figures 4A, 4C and 4E represent SNP distribution within each cohort, and Supplementary Figures 4B, 4D, and 4F represent SNP distribution between cohorts. The total number of ε4 carriers per ancestry were used to generate 4A and 4B. The total populations of each ancestry were used to generate 4C and 4D. The total numbers of ε4/ε4 carriers for each ancestry were used to generate 4E and 4F. Supplementary Figures 4B, 4D, and 4F show allele frequency ratios (AD-to-Control, left; Related dementias-to-Control, right) among APOE ε4 and APOE ε4/ε4 carriers for each of the candidate protective or disease-modifying variant, per ancestry. Warmer colors represent higher frequencies in cases versus controls, while cooler colors represent higher frequencies in controls versus cases, with dark blue (N/A) representing variants not present in either cases or controls. 0 values in cases and controls are shown in white.

E

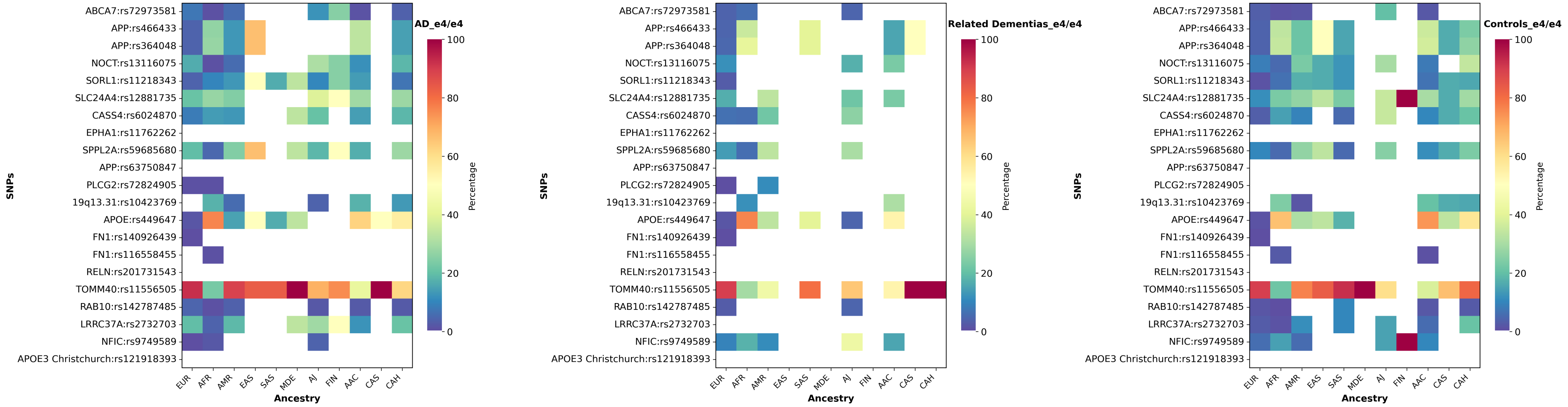

Supplementary Figure 4 - Proportions of individuals carrying both APOE ε4 or APOE ε4/ε4 genotypes and protective or disease-modifying variants across 11 genetic ancestries in Alzheimer's disease, related dementias, and controls in all datasets.

Supplementary Figures 4A, 4C and 4E represent SNP distribution within each cohort, and Supplementary Figures 4B, 4D, and 4F represent SNP distribution between cohorts. The total number of ε4 carriers per ancestry were used to generate 4A and 4B. The total populations of each ancestry were used to generate 4C and 4D. The total numbers of ε4/ε4 carriers for each ancestry were used to generate 4E and 4F. Supplementary Figures 4B, 4D, and 4F show allele frequency ratios (AD-to-Control, left; Related dementias-to-Control, right) among APOE ε4 and APOE ε4/ε4 carriers for each of the candidate protective or disease-modifying variant, per ancestry. Warmer colors represent higher frequencies in cases versus controls, while cooler colors represent higher frequencies in controls versus cases, with dark blue (N/A) representing variants not present in either cases or controls. 0 values in cases and controls are shown in white.

F

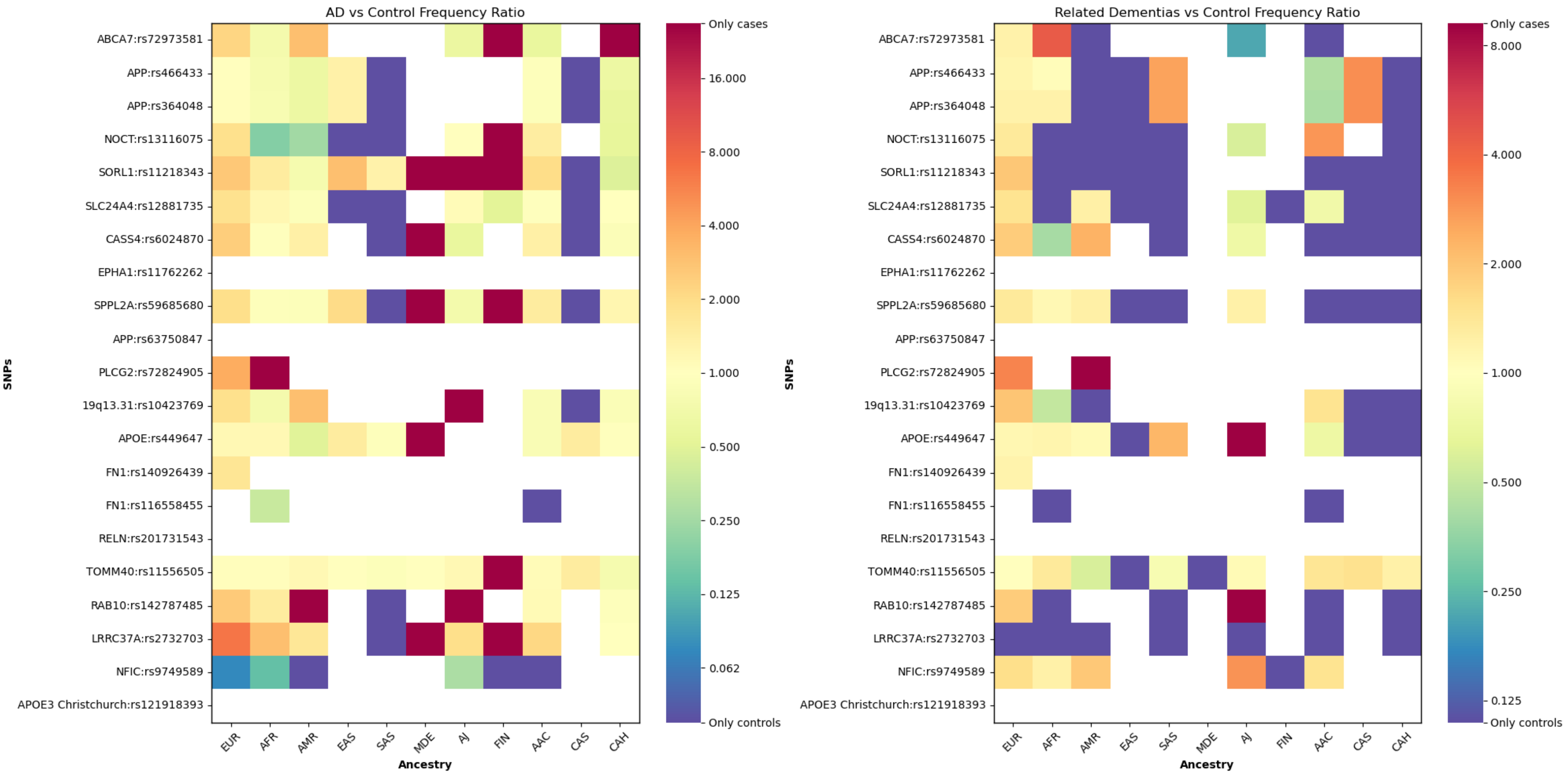

Supplementary Figure 4 - Proportions of individuals carrying both APOE ε4 or APOE ε4/ε4 genotypes and protective or disease-modifying variants across 11 genetic ancestries in Alzheimer's disease, related dementias, and controls in all datasets.

Supplementary Figures 4A, 4C and 4E represent SNP distribution within each cohort, and Supplementary Figures 4B, 4D, and 4F represent SNP distribution between cohorts. The total number of ε4 carriers per ancestry were used to generate 4A and 4B. The total populations of each ancestry were used to generate 4C and 4D. The total numbers of ε4/ε4 carriers for each ancestry were used to generate 4E and 4F. Supplementary Figures 4B, 4D, and 4F show allele frequency ratios (AD-to-Control, left; Related dementias-to-Control, right) among APOE ε4 and APOE ε4/ε4 carriers for each of the candidate protective or disease-modifying variant, per ancestry. Warmer colors represent higher frequencies in cases versus controls, while cooler colors represent higher frequencies in controls versus cases, with dark blue (N/A) representing variants not present in either cases or controls. 0 values in cases and controls are shown in white.

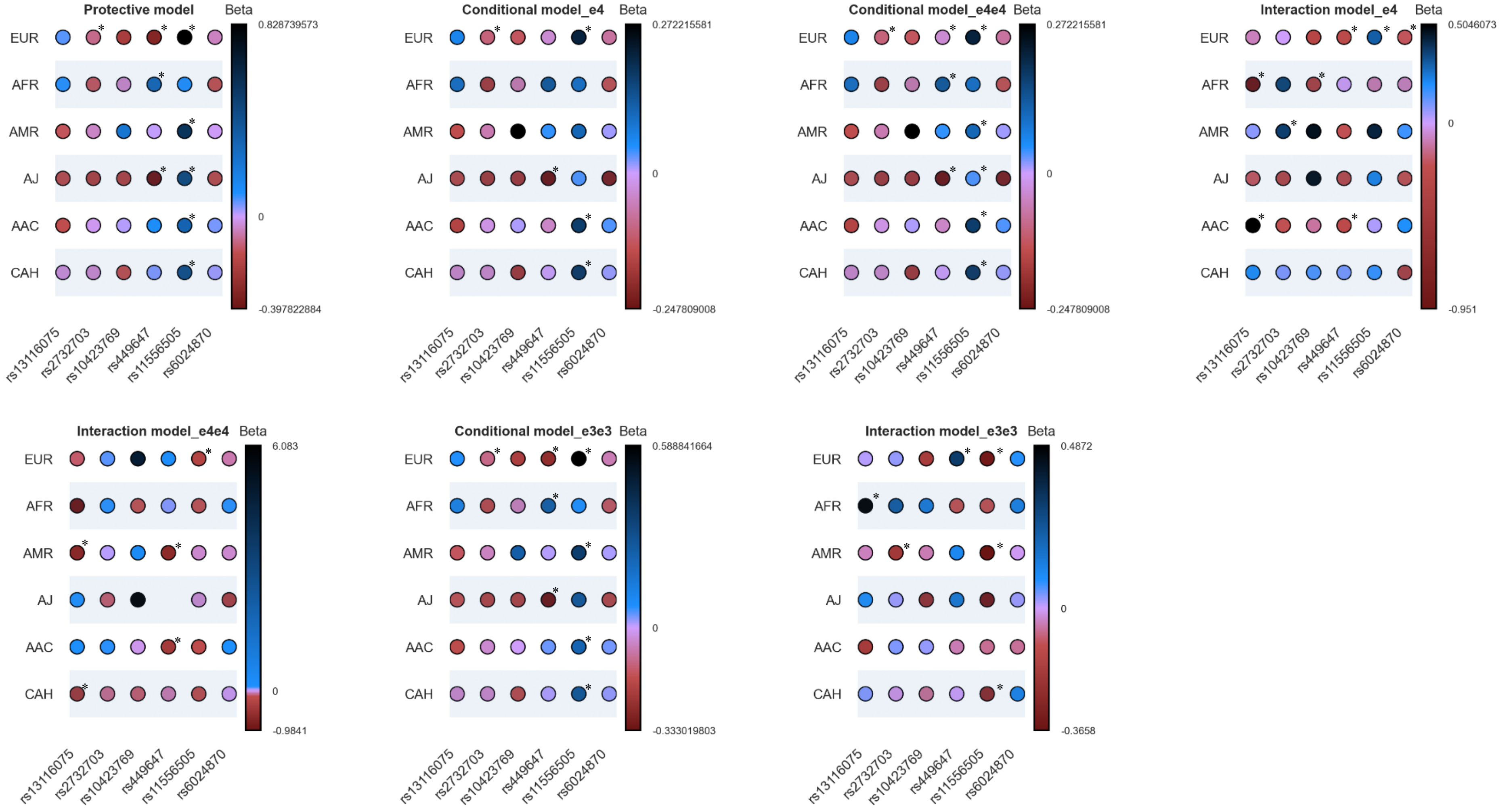

Supplementary Figure 5 - Upset plot showing protective, conditional, and interaction models across multiple ancestries.

The Y-axis represents each ancestry population with a large enough sample size, and the X-axis represents the six protective/disease-modifying variants. The color bar shows the magnitude of effects as log of the odds ratio (beta value) and directionality, with red color denoting negative directionality, and blue colors denoting positive directionality. * Indicates p-value < 0.05.

A

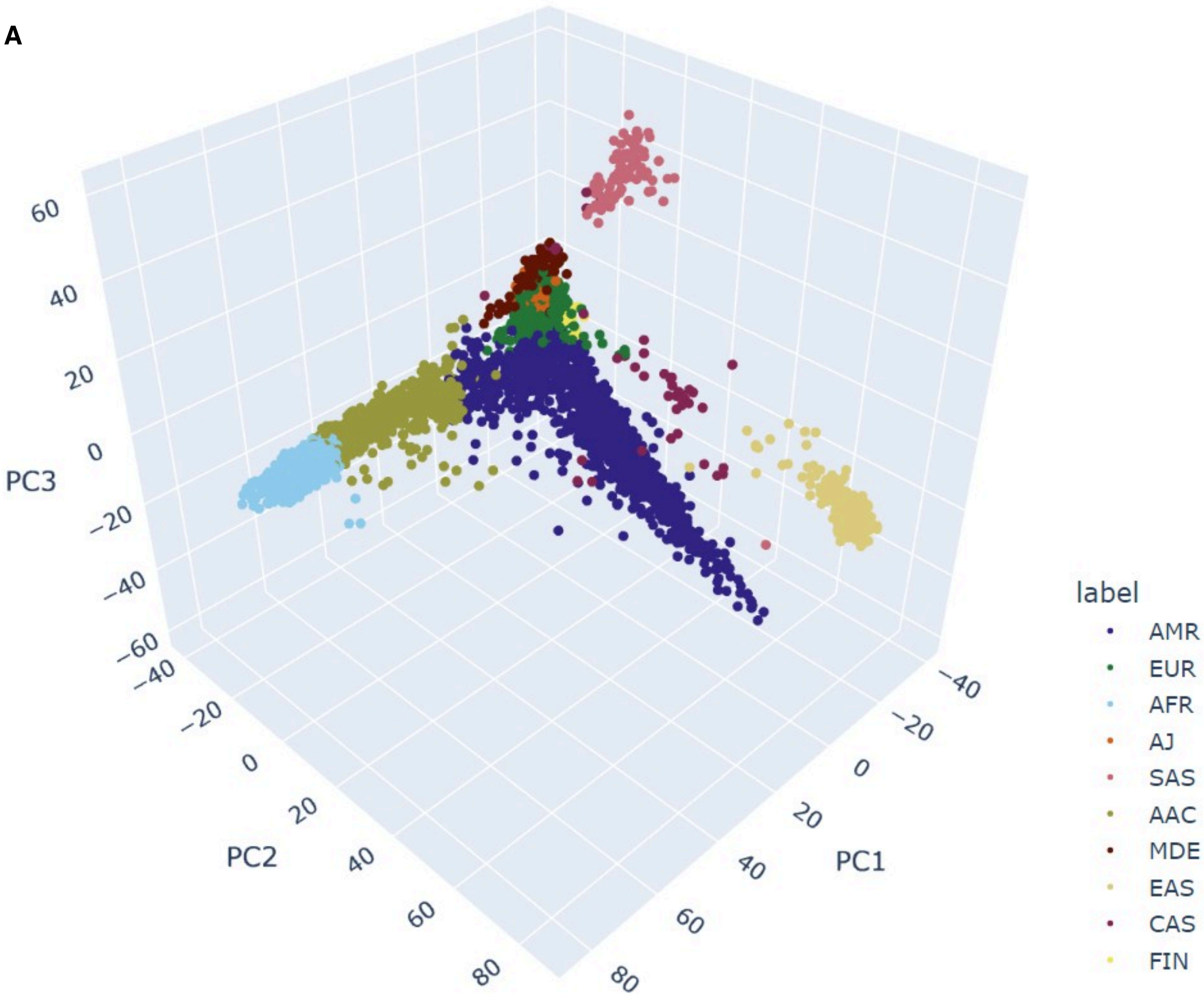

Supplementary Figure 6 - PCA plots in (A) All of Us, (B) UKB, (C) ADSP, (D) AMP PD, and (E) 100 KGP.

B

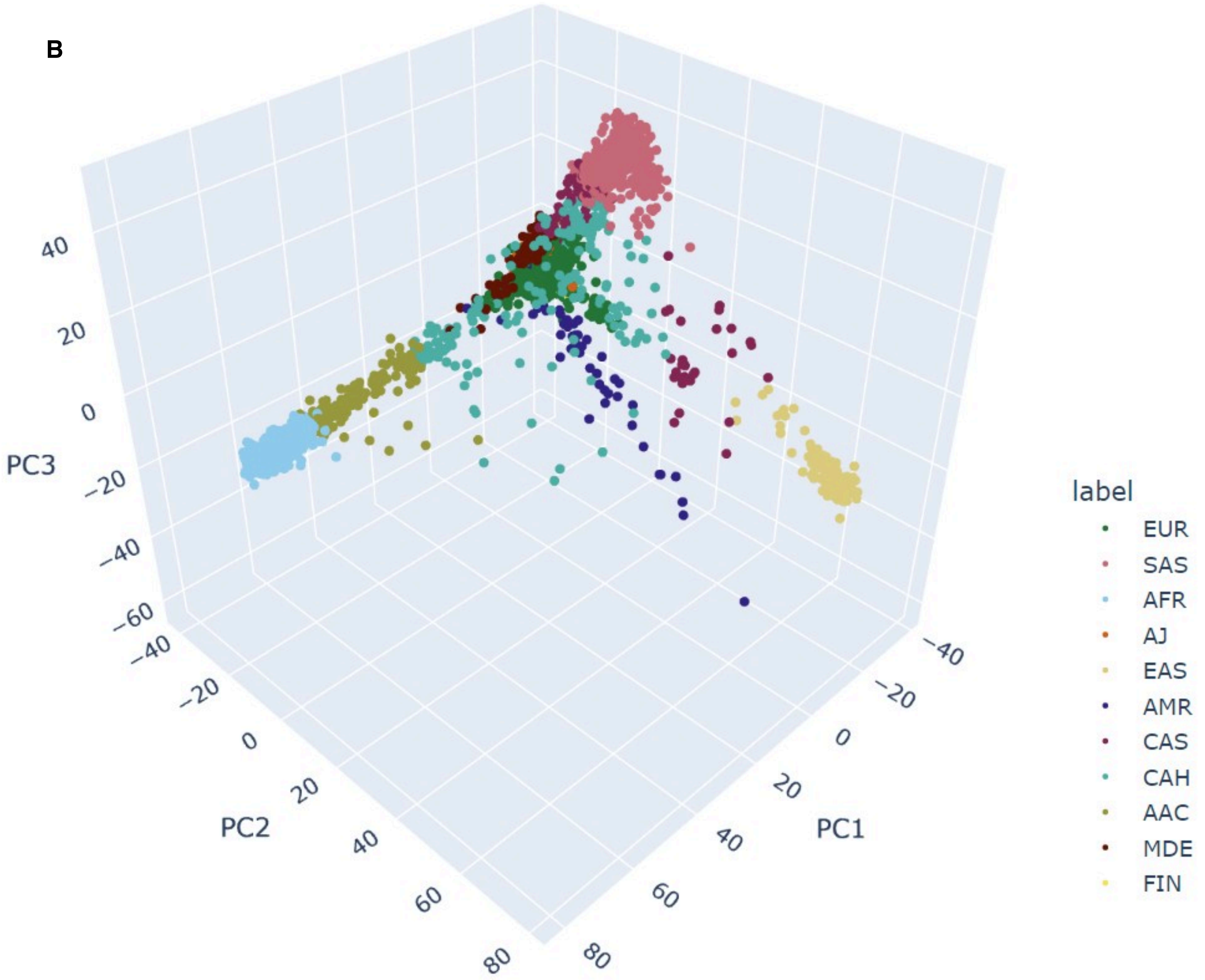

Supplementary Figure 6 - PCA plots in (A) All of Us, (B) UKB, (C) ADSP, (D) AMP PD, and (E) 100 KGP.

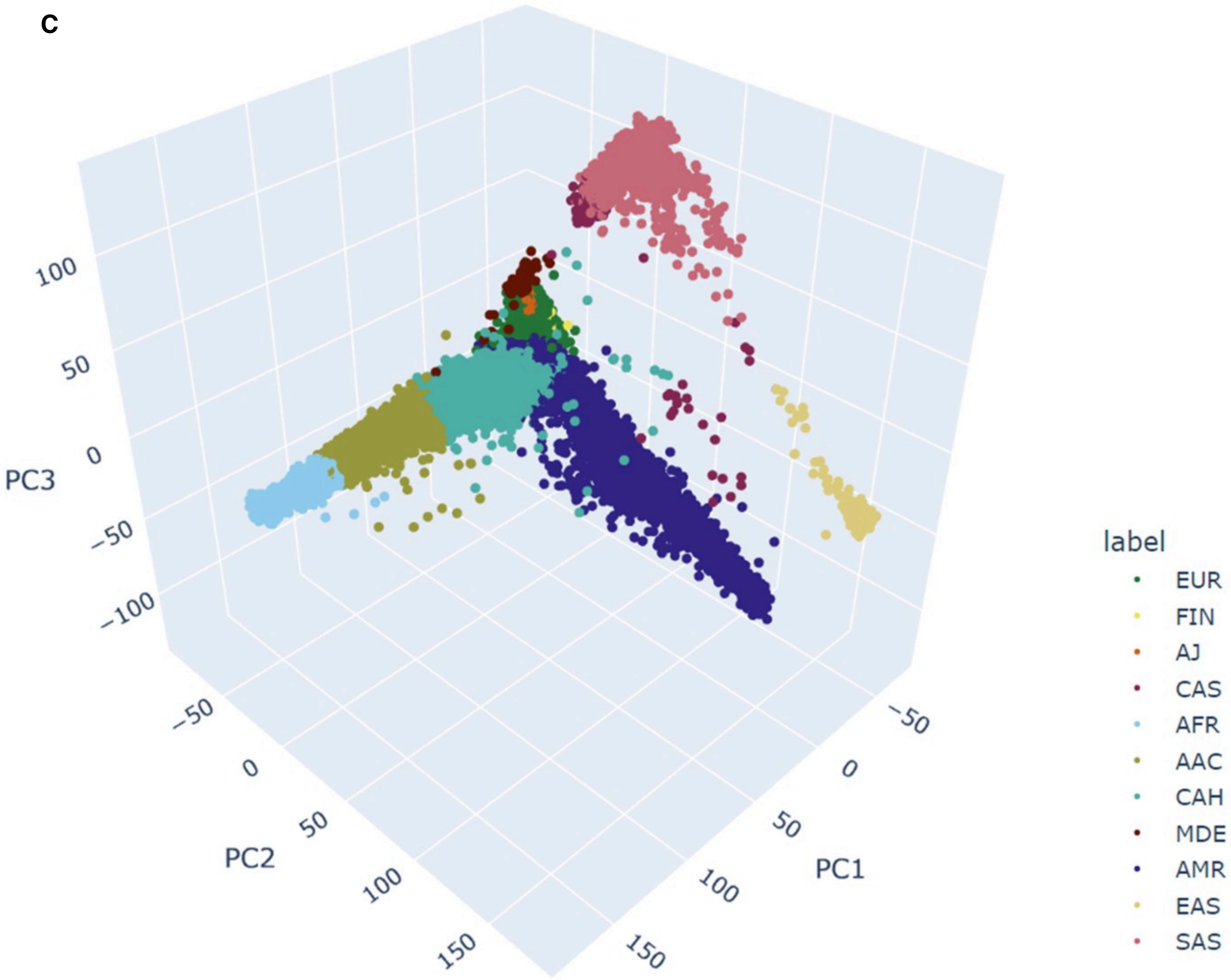

Supplementary Figure 6 - PCA plots in (A) All of Us, (B) UKB, (C) ADSP, (D) AMP PD, and (E) 100 KGP.

D

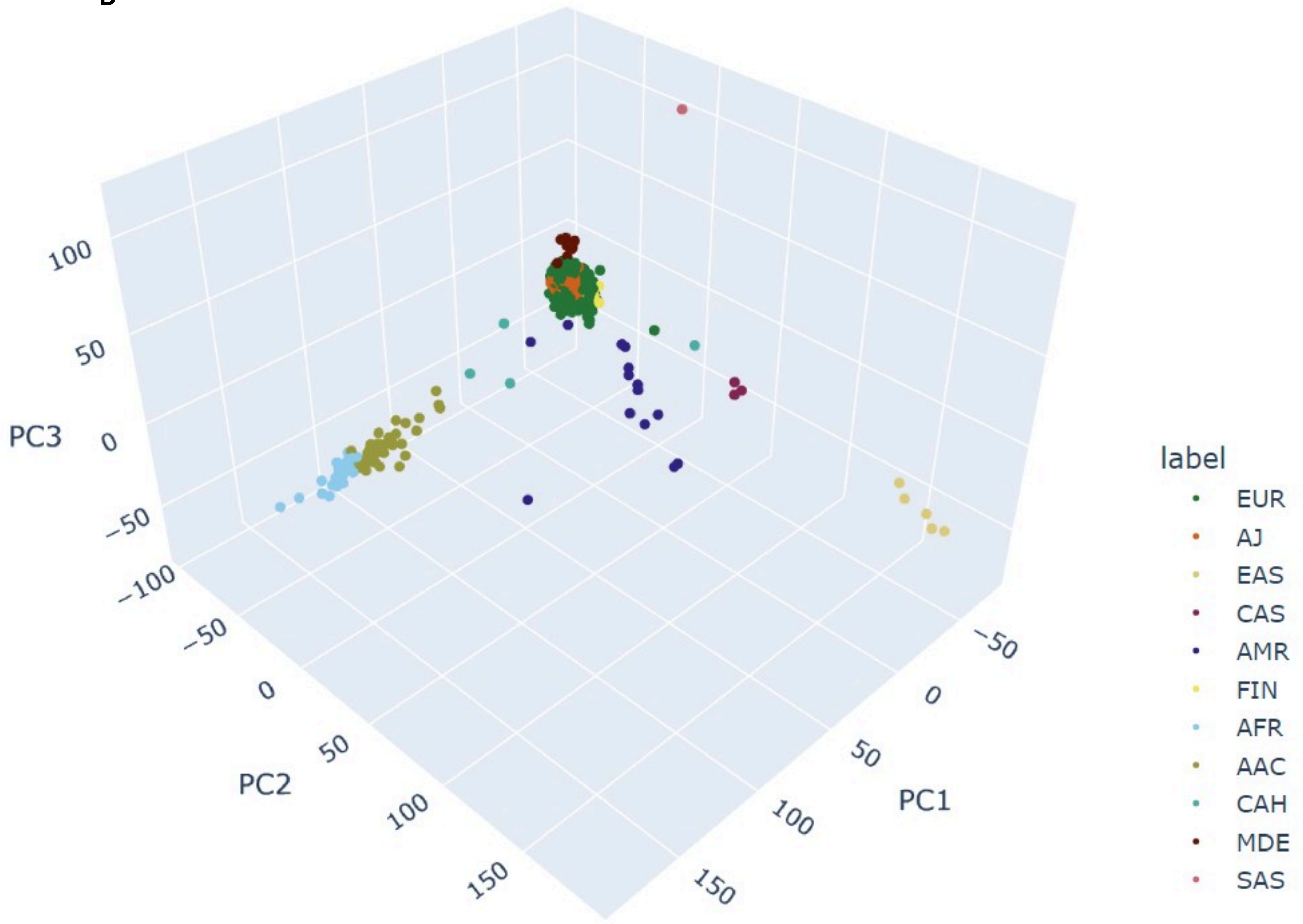

Supplementary Figure 6 - PCA plots in (A) All of Us, (B) UKB, (C) ADSP, (D) AMP PD, and (E) 100 KGP.

E

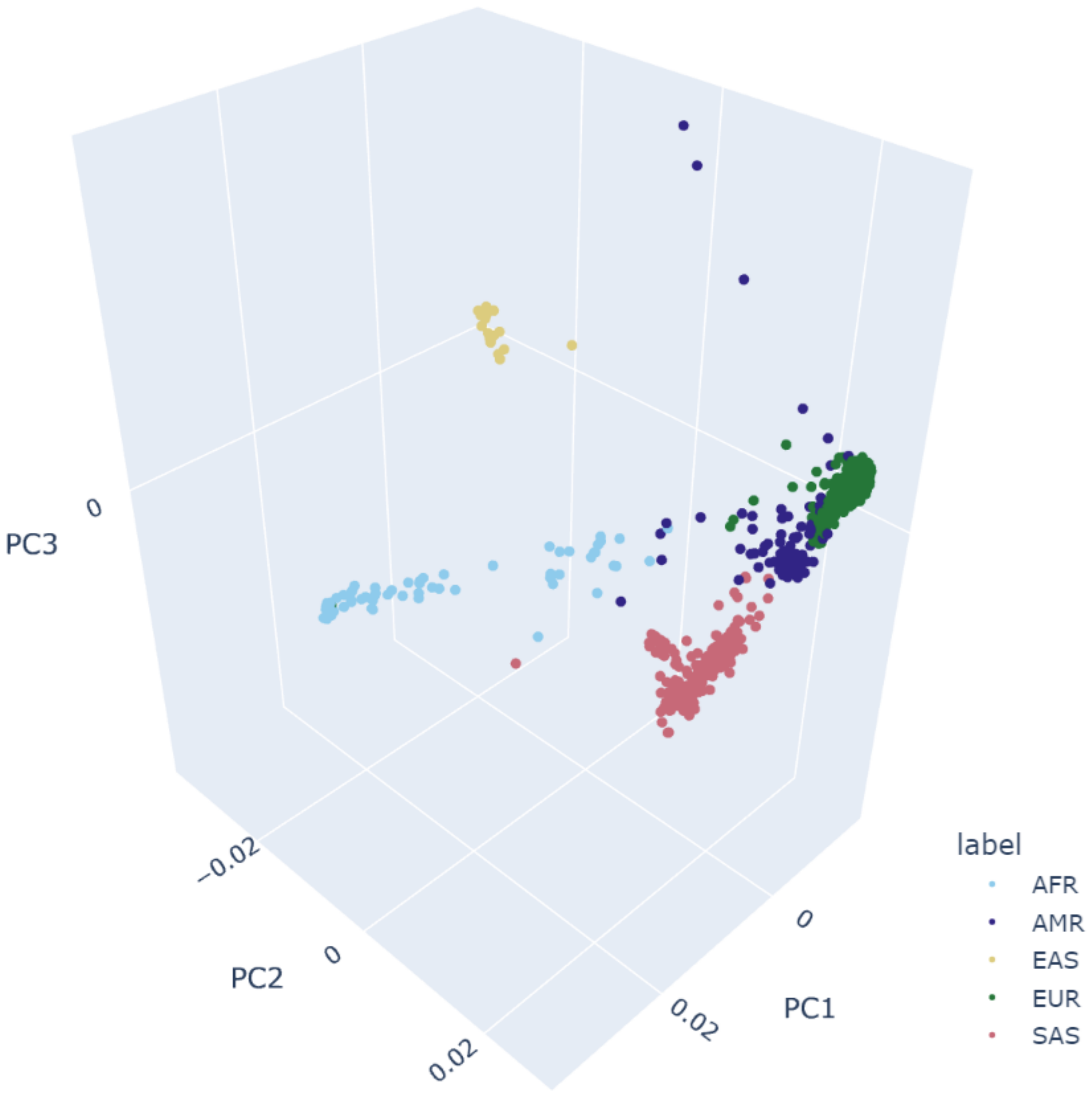

Supplementary Figure 6 - PCA plots in (A) All of Us, (B) UKB, (C) ADSP, (D) AMP PD, and (E) 100 KGP.
