# Supplementary material for: Biobank-scale genetic characterization of Alzheimer’s disease and related dementias across diverse ancestries
Source: Nat Commun. 2025 Aug 14;16:7554. doi: 10.1038/s41467-025-62108-y (PMC12354765; doi:10.1038/s41467-025-62108-y)
Supplement: Supplementary file 2 — Description of Additional Supplementary Files [file 41467_2025_62108_MOESM2_ESM.pdf]

Description of Additional Supplementary Files

Supplementary Data 1- Discovery phase: Multi-ancestry summary of all variants identified in Alzheimer's disease and related dementia cases in AoU

Supplementary Data 2- Discovery phase: Multi-ancestry summary of all variants identified in Alzheimer's disease and related dementia cases in UKB

Supplementary Data 3- Discovery phase: Multi-ancestry summary of all variants identified in Alzheimer's disease and related dementia cases in 100KGP

Supplementary Data 4- Replication phase: potential disease-causing variants only present in Alzheimer's disease and related dementia cases in ADSP

Supplementary Data 5- Replication phase: potential disease-causing variants only present in Alzheimer's disease and related dementia cases in AMP PD

Supplementary Data 6- Multi-ancestry summary of variants previously reported as potential disease-causing but identified in controls in multiple databases in this study

Supplementary Data 7- Phenotypic data for all individuals carrying known and novel potential disease-causing variants in the discovery phase

Supplementary Data 8- Phenotypic data for all individuals carrying known and novel potential disease-causing variants in the replication phase

Supplementary Data 9- Multi-ancestry summary of APOE genotypes in Alzheimer's disease and related dementia cases and controls in AoU, ADSP, UKB, AMP PD and 100KGP

Supplementary Data 10- Multi-ancestry summary of individuals carrying both APOE genotypes and protective or disease-modifying variants in patients and controls in AoU

Supplementary Data 11- Multi-ancestry summary of individuals carrying both APOE genotypes and protective or disease-modifying variants in patients and controls in ADSP

Supplementary Data 12- Multi-ancestry summary of individuals carrying both APOE genotypes and protective or disease-modifying variants in patients and controls in AMP PD

Supplementary Data 13- Multi-ancestry summary of individuals carrying both APOE genotypes and protective or disease-modifying variants in patients and controls in 100KGP

Supplementary Data 14- Multi-ancestry summary of individuals carrying both APOE genotypes and protective or disease-modifying variants in patients and controls in UKB

Supplementary Data 15- Combined results of data for individuals carrying both APOE genotypes and protective or disease-modifying variants in patients and controls across AoU, UKB, ADSP, 100KGP, and AMP PD

Supplementary Data 16- Assessment of the protective model in ADSP

Supplementary Data 17-Assessment of the conditional model for APOE ε4 carriers in ADSP

Supplementary Data 18- Assessment of the conditional model for APOE ε4/ε4 genotype in ADSP

Supplementary Data 19- Assessment of the correlation model for APOE ε4 carriers in ADSP

Supplementary Data 20-Assessment of the correlation model for APOE ε4/ε4 genotype in ADSP

Supplementary Data 21- Assessment of the interaction model for APOE ε4 carriers in ADSP

Supplementary Data 22- Assessment of the interaction model for APOE ε4/ε4 genotype in ADSP

Supplementary Data 23-Assessment of the conditional model for APOE ε3/ε3 genotype in ADSP

Supplementary Data 24- Assessment of the correlation model for APOE ε3/ε3 genotype in ADSP

Supplementary Data 25- Assessment of the interaction model for APOE ε3/ε3 genotype in ADSP

Supplementary Data 26- Results of Polygenic Risk Score and logistic regression for protective/disease-modifying variants in European ancestry in ADSP

Supplementary Data 27 – SKAT-O results of the burden analysis in European ancestry in ADSP

Supplementary Data 28 – Variants contributing to the burden analysis
